# Human Milk Oligosaccharides and Respiratory Syncytial Virus Infection in Infants

**DOI:** 10.1016/j.advnut.2024.100218

**Published:** 2024-04-05

**Authors:** Karina M Tonon, Somchai Chutipongtanate, Ardythe L Morrow, David S Newburg

**Affiliations:** Department of Environmental Health and Public Health Sciences, University of Cincinnati College of Medicine, Cincinnati, OH, United States

**Keywords:** antiviral, breastfeeding, gut microbiome, hMOS, immunomodulation, RSV

## Abstract

In infants worldwide, respiratory syncytial virus (RSV) is the leading cause of lower respiratory infections, including bronchiolitis, which is a major source of infant mortality. Bronchiolitis is the most common lower respiratory infection and the major cause of hospitalization in the first 6 mo of life. Infant responses to RSV infection are highly diverse, with symptoms varying from asymptomatic or mild to so severe as to require mechanical ventilation. Breastfed infants present a lower incidence and less severe forms of RSV lower respiratory infections. Among the multitude of human milk bioactive compounds, human milk oligosaccharides (hMOSs) are strong candidates for having a protective effect against RSV. hMOS reduces the viral load and the inflammatory signaling in cultured RSV-infected respiratory human cells. In addition to this direct effect, indirect mechanisms, notably gut microbiota composition and metabolism, have been proposed to mediate the protective effect of hMOS. Intake of infant formula containing synthetic hMOS has been shown to increase *Bifidobacterium* abundance and that of its metabolites, especially acetate, in infant feces and to reduce lower respiratory tract infections during the first year of life. Breastfeeding and the use of hMOS are promising approaches to protect against and treat RSV disease. Here, we review current evidence on the role of hMOS with regard to RSV infection and disease, attending to knowledge gaps and future research directions.

**Statement of significance**

Although the antiviral properties of human milk and human milk oligosaccharides have been reviewed, this review is focused on the less well-recognized roles of human milk oligosaccharides in preventing or attenuating respiratory syncytial virus infections and proposes possible mechanisms of protection.

## Introduction

Respiratory syncytial virus (RSV) is a pervasive viral pathogen that affects 90% of children aged <2 y. It is the most common cause of pediatric respiratory infection and the major cause of hospital admissions of infants worldwide [1]. RSV is a large enveloped, negative-sense, single-stranded RNA virus from the *Orthopneumovirus* genus of the Pneumoviridae family [2]. The G transmembrane protein (attachment glycoprotein) and F transmembrane protein (fusion protein) are the major antigens and critical for RSV virulence. The G protein mediates RSV attachment to the host respiratory epithelial cells, after which the F protein enables fusion of the host plasma membrane and viral envelope membrane and capsid to permit viral genes passage into the host cell. The F protein also promotes the aggregation of cells through the fusion of their plasma membranes, producing the cognate multinucleated syncytia and allowing the transmission of virus from cell to cell. RSV has 2 major antigenic strains, RSV-A and RSV-B, which have distinct epitopes in the F and G proteins in addition to several subtypes [3,4]. RSV-A and RSV-B can cocirculate with alternating dominance annually. Although there are some reports linking RSV-A to higher morbidity, it is not consistently observed among studies [3].

**FIGURE 1:**
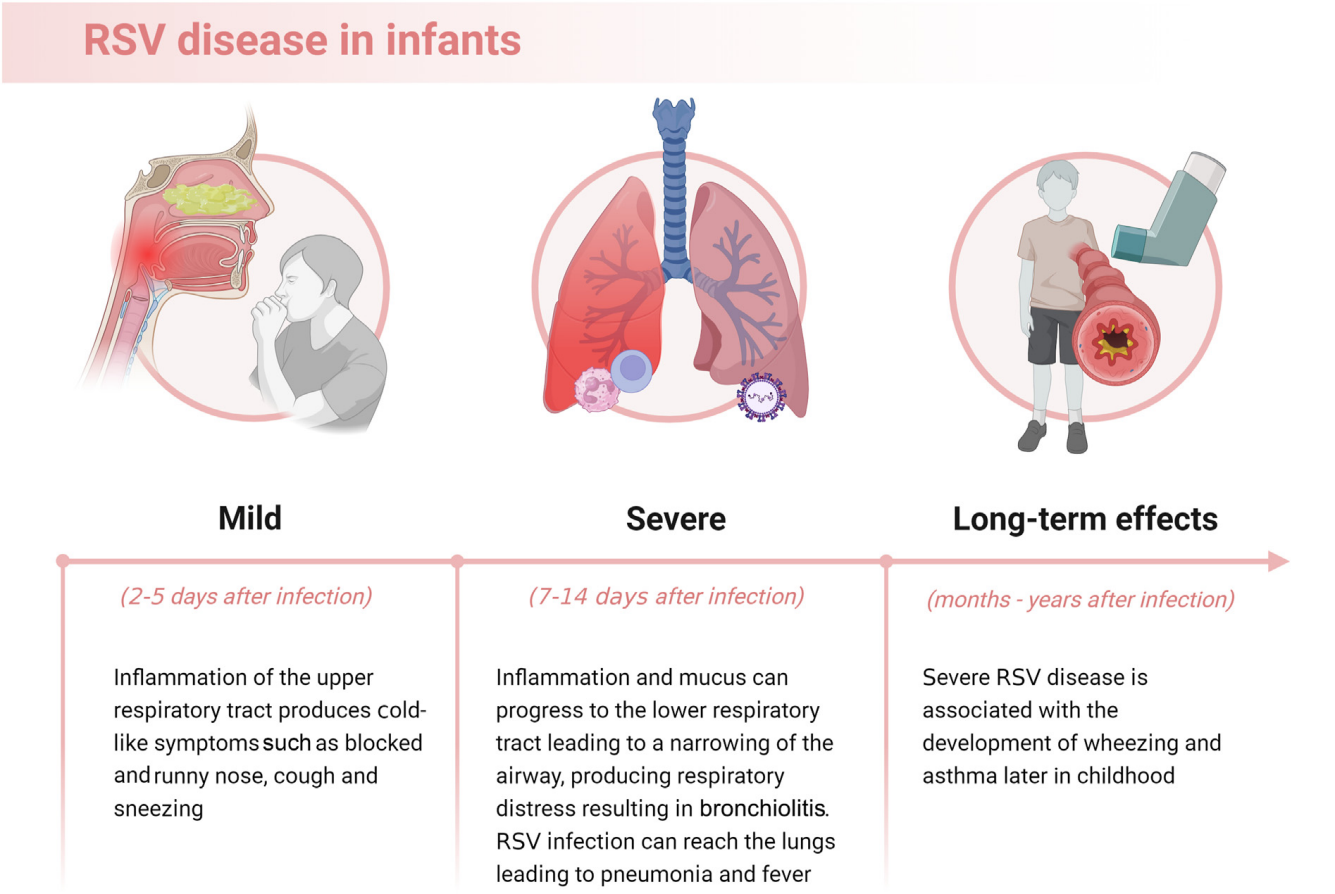
Progression of RSV disease in infants. RSV, respiratory syncytial virus.

A substantial proportion of RSV-associated morbidity occurs in the first year of life. Of all hospital admissions and deaths due to RSV disease, 45% occur in children aged <6 mo [1,5]. Globally, 1.4 million hospital admissions, 13,300 in-hospital deaths, and 45,700 RSV-attributable overall deaths occurred in this age group in 2019 [5]. Morbidity and mortality due to RSV infection are higher in premature infants and in infants with chronic lung disease or hemodynamically significant congenital heart disease [6]. RSV transmission occurs through inoculation of the nasopharyngeal or conjunctival mucosa with droplets or dry respiratory secretions, such as those present on bedclothes and hard surfaces from infected individuals or skin from infected or noninfected persons. Clinical manifestations of RSV infection vary strikingly, from asymptomatic or mild symptoms to severe disease. Typically, the infection starts with signs and symptoms of mucosal inflammation and irritation of the upper respiratory tract, manifesting as a congested and runny nose and sneezing. Symptoms can progress to the lower respiratory tract, manifesting as a cough and respiratory distress typical of bronchiolitis (Figure 1) [6]. RSV accounts for ≤80% of bronchiolitis in infants. Death from bronchiolitis occurs disproportionately in low-income countries, and bronchiolitis is the leading cause of infant hospitalization in high-income countries [7]. RSV disease impacts the quality of life and imposes an immense financial burden on the family and the health care system [8,9].

In addition to the burden of acute disease, RSV infection may influence immune development and long-term health. Respiratory symptoms such as wheezing and coughing can persist after the infection resolves. Children with a history of RSV infection have ≤3 times more recurrent wheezing episodes than children without an RSV history [10]. Severe RSV disease in early life is associated with an increased risk of asthma later in childhood, especially in infants with allergic predisposition [11]. Asthma is more frequent in young adults who were hospitalized for bronchiolitis during infancy [25.1%; 95% confidence interval (CI): 19.0, 31.2%] than those who had not been hospitalized [13.1%; 95% CI: 7.9, 18.2%, *P* ≤ 0.05], irrespective of RSV or other etiology [12]. RSV infection induces a strong systemic and pulmonary mucosal immune response, beginning with potent neutrophil activation followed by systemic T-cell lymphopenia. At the end of the acute phase, an increase in pulmonary CD8+ T-cell response mediates viral clearance and recovery from disease [13]. Nonetheless, RSV infection induces a weak adaptive immune response, allowing recurrent infections in infancy and later life [13]. Infants with RSV infection also have a T-helper 2-biased immune response that may play an important role in asthma pathophysiology [14]. Although an enhanced T-helper 2 response is commonly reported in RSV-mediated atopic asthma, the RSV mechanism in the onset of nonatopic asthma is less clear [15]. Although associations are reported, a causal relationship between RSV infection in early life and asthma later in childhood has not been proven [16].

Given the acute and long-term disease burden RSV infection imposes on infants and the dearth of effective treatments, prophylaxis strategies are an urgent need. An RSV vaccine, Abrysvo (Pfizer), has recently been approved as maternal immunization to prevent severe RSV infection in newborns ≤6 mo of age [17]. However, for infants whose mothers did not receive RSV vaccine during pregnancy, prevention is currently limited to passive immunization by monoclonal antibodies, nirsevimab, or palivizumab, which is only indicated for infants who are at increased risk of severe RSV disease [18,19]. Although nirsevimab and palivizumab have acceptable effectiveness, these monoclonal antibodies are expensive and mostly available to high-income countries [[19], [20], [21]]. In contrast, breastfeeding, a universal primary prevention strategy to reduce infectious diseases in infants, consistently confers benefits against both the incidence and severity of RSV bronchiolitis in infants [22]. A systematic review of 19 studies comprising 16,787 infants from 31 countries concluded that not being breastfed poses a significant risk of severe RSV disease and hospitalization [22]. Conversely, exclusive breastfeeding for 4–6 mo significantly reduces hospitalization, length of stay, supplemental oxygen therapy, and admission to intensive care units [22].

The protection conferred by breastfeeding is consistent with a mother-infant molecular link through breast milk. Human milk contains high concentrations, types, and diversity of bioactive components that can participate in an immune response against viral infections, including Igs, immune cells, cytokines, chemokines, and oligosaccharides [23]. However, the role of these molecules in preventing or ameliorating RSV infection has not been defined. Recently, associations between human milk oligosaccharides (hMOS) and lower risk of respiratory infections in infants aged ≤12 mo, as well as reduced viral load and inflammatory response, have been reported [[24], [25], [26]]. Here, the current evidence about the preventive effects of hMOS against RSV infection is reviewed.

### Overview of hMOS

**FIGURE 2:**
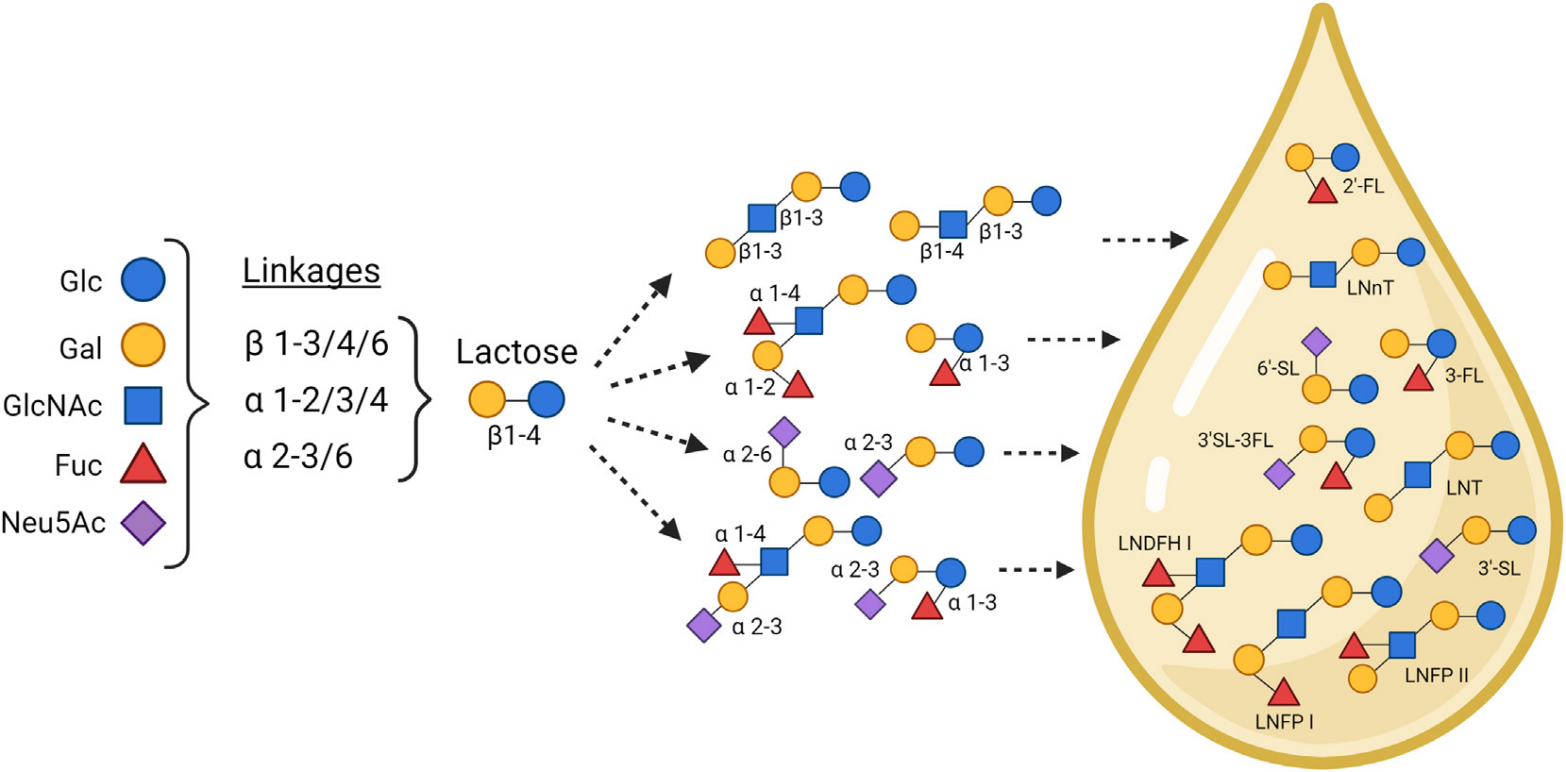
Relevant hMOS and their biosynthesis in the mammary gland. Fuc, fucose; Gal, galactose; Glc, glucose; GlcNAc, N-acetyl-glucosamine; hMOS*,* human milk oligosaccharides; LNDFH I, lacto-N-difucosyl-hexaose I; LNFP I, lacto-N-fucopentaose I; LNFP II, lacto-N-fucopentaose II; LNnT, lacto-N-neotetratose; LNT, lacto-N-tetraose; Neu5Ac, N-acetylneuraminic acid; 2’-FL, 2’-fucosyllactose; 3-FL, 3-fucosyllactose; 3’-SL, 3’-sialyllactose; 3’-SL-3’-FL, 3’-sialyl-3-fucosyllactose; 6’-SL, 6’-sialyllactose.

hMOS are highly abundant unconjugated glycans in human milk, and they play a multifunctional role in infant development [27]. hMOS are produced in the mammary gland from a lactose molecule, which can be elongated with N-acetyl-glucosamine (GlcNAc) and galactose (Gal) to form type 1 (Galβ1-3GlcNAc-lactose; lacto-N-biose-lactose) or type 2 (Galβ1-4GlcNAc-lactose; lactosamine-lactose) chains with additional β1-3 (linear) or β1-6 (branched) linkages to produce the core structures. Moreover, fucose (Fuc) and N-acetyl-neuraminic acid (Neu5Ac, sialic acid) can be attached to the hMOS core or directly to lactose to produce fucosylated and sialylated structures or those containing both moieties (Figure 2) [28,29]. Certain hMOS mimic ABO and Lewis (Le) histo-blood group antigens present on the surface of red blood and mucosal epithelial cells, as well as in secretions [30]. Altogether, the hMOS fraction ranges in total concentration from <5 to 20 g/L and comprises the third most abundant solid component of human milk, after lactose and lipids [[31], [32], [33], [34], [35]]. More than 160 distinct molecules have been identified to date within the hMOS fraction [36].

The relative abundance and concentrations of hMOS vary significantly among populations of women; they also vary within milk samples from individual women over the course of lactation [37,38]. The population variations are caused by genetic factors, especially the relative activity of the FUT2 and FUT3 genes involved in hMOS biosynthesis within the mammary gland [35,39,40]. FUT2 encodes the enzyme α1-2 fucosyltransferase, which attaches a Fuc to the lactose or hMOS chain in an α1-2 position, whereas FUT3 encodes α1-3/4 fucosyltransferase, an enzyme that links a Fuc to the lactose or to the hMOS chain in an α1-4 position [41,42]. When mutations occur in 1 or in both genes, human milk lacks or has lower amounts of α1-2 or α1-4 fucosylated hMOS [43]. Besides genetics, environmental and maternal factors such as geographic location, BMI (in kg/m^2^), and parity may also be associated with hMOS concentrations [37,44].

The diversity in structural complexity and abundance grants multiple properties to the hMOS. hMOS are not well digested by the infant. Between 1% and 5% of hMOS may be absorbed into the circulation; thus, ∼95 to 99% reach the colon intact [45,46]. hMOS in the gut modulates the microbiome, favoring the growth of mutualistic bacteria, especially *Bifidobacterium spp* and *Bacteroides spp* [47]. The hMOS in the gut also prevents pathogen binding to the intestinal epithelial cells, acting as competitive inhibitors (soluble decoys) to inhibit pathogens binding to their targets in the infant gut mucosa. hMOS also reduces the inflammatory response in mucosal epithelial cells exposed to pathogens, promoting intestinal homeostasis, including maintenance of the gut barrier [[48], [49], [50]]. Another remarkable feature is their modulation of the immune system through the interaction with immune cells, acting as signaling molecules [51,52]. All of these hMOS mechanisms could participate in inhibiting viral infections and disease in breastfed infants.

### hMOS lowers the risk of respiratory infections

An association between hMOS consumption and respiratory symptoms in infants was reported in 2006 by Stepans et al. [53]. Infants who experienced runny nose, cold, cough, or pneumonia by 3 mo of age had significantly lower concentrations of lacto-N-fucopentaose II (LNFP II) in their mother’s milk at 2 wk of age (6.6 ± 1.6 μg/mL) than did infants without respiratory symptoms (9.0 ± 1.5 μg/mL; *P* = 0.03). Infants who had respiratory symptoms by 6 wk had significantly lower concentrations of LNFP II in their feces at 2 wk of age (350 ± 80 μg/g) than nonsymptomatic infants (770 ± 90 μg/g, *P* = 0.01) and infants who had respiratory symptoms by 3 mo had significantly lower concentrations of LNFP II in their feces (580 ± 110 μg/g) than nonsymptomatic infants (840 ± 100 μg/g) [53]. By logistic regression analysis, LNFP II concentration in mother’s milk at 2 wk postpartum was a predictor of infant respiratory problems at 6 wk [odds ratio (OR): 0.67; 95% CI: 0.46, 0.99; *P* = 0.01] and 3 mo of age (OR: 0.80; 95% CI: 0.62, 1.03; *P* = 0.04), whereas LNFP II in infant feces was a significant predictor of respiratory symptoms only at 6 wk (OR: 0.576; 95% CI: 0.34, 0.99; *P* = 0.003) [53]. The study suggests an association between a lower milk concentration (hence intake) of LNFP II at 2 wk and a higher incidence of respiratory problems in the first months of life. LNFP II contains the Lea antigen (Galβ1-3(Fucα1-4)GlcNAcβ1-3Galβ1-4Glc) with potential antiviral activity. hMOS resembling other adhesion sites of respiratory pathogens could be investigated for inhibition of other pathogens in future studies.

Clinical studies suggest that other hMOS can protect against respiratory diseases. Puccio et al. [24] (2017) assessed whether infant formula containing the hMOS 2’-fucosyllactose (2’-FL) and Lacto-N-neotetraose (LNnT) affects infant growth and morbidity. Healthy infants ≤14 d old received for 6 mo either a tested formula with 1.0 g/L 2’-FL and 0.5 g/L LNnT (*n* = 88) or a control formula (*n* = 87) of the same composition but without 2’-FL and LNnT. They were followed up until they reached 1 y old. Infants fed the formula with 2’-FL and LNnT had fewer cases of parent-reported lower respiratory tract infections (LRTIs) (a disease cluster including bronchitis, pneumonia, RSV bronchiolitis, among others) during the first year of life (19%; *n* = 17) compared to infants fed the control formula (35%; *n* = 30) (OR: 0.45; 95% CI: 0.21, 0.95; *P* < 0.05) [24]. Bronchitis during the first year of life was also reported significantly less in the infants fed hMOS formula (10%; *n* = 9) than in the infants fed the control formula (28%; *n* = 24) (OR: 0.30; 95% CI: 0.11, 0.73; *P* ≤ 0.01); antibiotic use was also less (42%; *n* = 37) in the hMOS population than in those fed the control formula (61%; *n* = 53) (OR: 0.47; 95% CI: 0.24, 0.89; *P* < 0.05) [24]. Infants born by cesarean section and receiving the hMOS formula (*n* = 32) had even more pronounced differences in the occurrence of LRTIs from those (*n* = 32) receiving control formula (13% compared with 41%; OR: 0.21; 95% CI: 0.04, 0.83; *P* = 0.022) and bronchitis (3% compared with 34%; OR: 0.21; 95% CI: 0.04, 0.83; *P* = 0.022) [24]. As infants consumed 2’-FL and LNnT only during the first 6 mo of life but presented lower reports of respiratory disease throughout the whole first year, these data suggest long-lasting protection by hMOS consumption.

A prospective cohort study in Bangladesh compared the incidence of acute respiratory infections (ARIs) during the first 2 y of life in breastfed infants according to maternal secretor (Se) and Le genotypes as proxies of hMOS in breast milk [54]. Twenty-four (11%) of the 210 mothers were non-Ses. Maternal Se genotype was associated with reduced ARIs from birth to 6 mo of age, but not after, with an incidence rate ratio of 0.66 (95% CI: 0.47, 0.94; *P* = 0.02). The maternal Le genotype was not associated with ARIs at any time point between birth and 2 y. When analyzed during the exclusively breastfeeding period of birth to 4 mo of age, which coincided with the peak of ARIs in the study population, the infants from Se mothers presented a lower occurrence of ARIs (OR: 0.34, 95% CI: 0.13, 0.92; *P* = 0.03). The infant nasopharyngeal microbiome was not associated with the maternal Se or Le genotype, suggesting that the protective effects of hMOS are not mediated through changes in the nasopharyngeal microbiome. The association between the maternal Se genotype and ARIs suggests a protective effect of the α1-2 fucosylated hMOS against respiratory infections, consistent with the clinical findings of 2’-FL infant formula. However, hMOS concentrations vary significantly among Se mothers and may have a dose-dependent effect. Maternal Se genotype also impacts constituents of breast milk other than hMOS, such as glycoproteins and the maternal microbiome, with a possible impact on the infant.

Some studies did not find associations between hMOS and a lower risk of respiratory infections. In a birth cohort in Germany, Siziba et al. [55] (2021) investigated associations between 15 individual hMOS concentrations at 6 wk postpartum with the prevalence of infections in 667 infants (75% exclusively breastfed) ≤2 y of age. The prevalence of LRTIs was slightly higher in non-Se milk-fed infants than in Se milk-fed infants in their first year of life (35% compared with 28%) as well as in their second year (50% compared with 43%). However, no significant associations appeared between any individual or total hMOS and LRTIs, such as bronchitis, bronchiolitis, and pneumonia, in their medical records after correcting for multiple variables [55]. In a cohort of 647 Malawian mother-infant pairs receiving micronutrient or lipid supplementation, Jorgensen et al. [56] (2021) compared concentrations of 51 hMOS at 6 mo postpartum and the incidence of ARIs in infants at 6–12 mo of age. ARIs were reported by mothers and defined by cough, rapid or difficult breathing, and nasal discharge. The several positive and negative associations from an exploratory analysis between diverse hMOS and ARIs proved to be not significant after correction for multiple testing [56]. The intervention with nutritional supplements could also have contributed to milk production, composition, and infant outcomes, thereby confounding any innate relationship between milk components and infant health. Table 1 [24,[53], [54], [55], [56]] provides a summary of the clinical studies linking hMOS, RSV, and/or respiratory infections.

**TABLE 1:** Summary of the clinical studies on human milk oligosaccharides, respiratory syncytial virus, and/or respiratory infections

Study type and design	Virus/outcome	Respiratory infections assessment	hMOS	Major results	Reference
Clinical (randomized, double-blinded, controlled trial)	Primary outcome was weight gain. Secondary outcomes included the occurrence of respiratory disease and medication use	Parental reports	2’-FL and LNnT	Infants who received an infant formula with 2’-FL and LNnT until 6 mo old presented fewer episodes of bronchitis, LRTIs, and antibiotics use during the whole first year of life than controls	[24]
Observational (case-control)	Respiratory symptoms or pneumonia	Maternal reports	LNFP II	Infants who experienced respiratory problems in the first months of life had lower LNFP II concentrations in the feces and in mother’s milk at 2 wk of age than infants who did not have respiratory problems	[53]
Observational (prospective cohort)	Incidence of ARIs in the first 2 y of life	Active surveillance^1^	Maternal *Se* and *Le* genotypes as proxies of fucosylated hMOS	Maternal *Se* genotype was associated with reduced ARIs from birth to 6 mo of age (IRR: 0.66; 95% CI: 0.47–0.94; *P* = 0.02).	[54]
Observational (prospective cohort)	Prevalence of respiratory infections ≤2 y of life	Medical records	15 individual hMOS and hMOS total concentrations in mother’s milk	No associations were observed between any of the studied hMOS or hMOS total concentration and respiratory infections	[55]
Observational (sub-study of a clinical trial with maternal nutrient supplementation)	Incidence of ARIs from 6 to 12 mo of age	Maternal reports	51 individual hMOS, hMOS classes, and total hMOS in mother’s milk	No significant associations were found after correction for multiple testing between any of the studied hMOS, hMOS classes, or hMOS total concentrations and ARIs.	[56]

ARI, acute respiratory infection; CI, confidence interval; hMOS, human milk oligosaccharides; IRR, incidence rate ratio; Le, Lewis; LNFP II, lacto-N-fucopentaose II; LNnT, lacto-N-neotetraose; LRTI, lower respiratory tract infection; Se, secretor; 2’-FL, 2’-fucosyllactose.

1 The authors do not provide details about the active surveillance of ARIs.

These reports suggest that future studies of larger cohort size, an assessment of respiratory infections that include viral etiology and disease severity, and precise assessment of the composition and concentrations of various hMOS in mother’s milk, maternal and infant Se status, and microbiome, would be promising to better understand any association between hMOS consumption and RSV incidence and severity.

### Antiviral activity of hMOS

Some hMOS can bind clinically relevant viruses, such as norovirus, rotavirus, influenza, and HIV [57]. For example, α1-2 fucosylated hMOS, such as 2’-FL, can occupy the binding sites of common norovirus strains [58,59], and above-average concentrations of lacto-N-difucohexaose I are associated with a lower incidence of norovirus diarrhea in infants [60]. A major cause of severe gastroenteritis in neonates and infants is rotavirus [61]. Sialylated or nonsialylated glycans can serve as binding sites for human rotavirus in a strain-dependent manner [62,63]. Although 2’-FL is more efficient in reducing rotavirus G1P[8] strain infectivity, 3’-sialyl-lactose (3’-SL) and 6’-sialyl-lactose (6’-SL) are more effective against rotavirus strain G2P[4] [63]. On the contrary, human neonate-specific rotavirus G10P[11] strains bind to both lacto-N-tetraose (LNT) and LNnT [62]. Curiously, 2’-FL and LNT increase the G10P [11] infectivity in vitro, and higher concentrations of 2’-FL, LNT, and 6’-SL in breast milk are associated with symptomatic rotavirus infection in neonates [64]. Influenza viruses bind to sialylated motifs, with avian strains binding preferentially to α2-3 sialic acid, whereas human strains have a strong preference for α2-6 sialic acid [65]. Both 6’-SL and LNnT reduce influenza infection in human respiratory epithelial cells, possibly acting as competitive binding inhibitors [66].

Besides binding to viruses, hMOS can compete for their binding sites in the host cell. Several hMOS containing Le determinants compete with the HIV-1 virus for the binding site at the dendritic cell on the intestinal mucosa, reducing its binding to cultured cells by 80% in a dose-dependent manner[67,68]. Higher hMOS total concentrations in human milk from HIV-infected women were associated with lower transmission to the infant in a nested case-control study [69].

There is an interesting contradiction between human milk and viral infections. Although human milk can transmit viruses to infants, it rarely causes disease in them. This may be because of the antiviral properties of human milk, which were brilliantly explained in a recent review [73]. Different hMOS bind to different viruses and viral genotypes. Thus, the structural diversity of hMOS can provide the infant with a wide range of protection from infections. The emergence of the COVID-19 pandemic increased the enthusiasm for the potential of human milk and hMOS to prevent viral infections, as denoted by reviews on the subject [57,70,71,72,73]. Despite RSV being the major cause of respiratory infections in infants, the ability of hMOS to inhibit RSV infection and pro-inflammatory responses to infection is understudied compared to other viruses; the 2 studies to date are discussed below.

### hMOS alters host innate response to prevent RSV infection

Exposing human respiratory and peripheral blood mononuclear cells (PBMCs) to hMOS alters their response to RSV infection, whereas Fuc, lactose, or the prebiotic galacto-oligosaccharides have no effect. In 16HBE respiratory cells derived from a 1-y-old infant, Duska-McEwen et al. [66] (2014) demonstrated that 2’-FL and 3’-SL at 50 μg/mL or below significantly decrease RSV viral load and cytokines associated with RSV disease severity (IL-6, IL-8, and macrophage inflammatory protein-1α) and inflammation (TNF-α and monocyte chemoattractant protein-1). 2’-FL at concentrations from 1 to 10 μg/mL reduced RSV viral load, and 6’-SL at 1000 μg/mL reduced IL-6 expression in Calu-3 respiratory cells derived from a 25-y-old adult [66]. In RSV-infected adult PBMCs, 6’-SL had no effect on viral load but down-regulated interferon gamma-induced protein 10 and TNF-α in a dose-dependent manner. LNnT did not affect viral load or cytokine expression in airway epithelial cells or PBMCs after RSV infection [66].

In a sub-study of a randomized, double-blinded, controlled trial to test the safety of infant formulas with 2’-FL, a similar ability of 2’-FL to decrease inflammatory response was reported by Goehring et al. [26] (2016). Infants who received experimental formulas containing 0.2 or 1.0 g/L 2’-FL presented significantly lower plasma concentrations of the inflammatory cytokines TNF-α, IL-1α, IL-1β, and IL-6 than infants fed the control formula without 2’-FL, and similar to concentrations seen in breastfed infants. When challenged with RSV to simulate an infection, PBMCs from infants who received the formula with 0.2 g/L 2’-FL expressed significantly lower concentrations of TNF-α, IL-1β, and IL-6 compared with the control group but similar concentrations to the breastfed infants [26]. RSV viral load on PBMCs did not differ between the groups, as in the study by Duska-McEwen et al. [66] (2014), where 2’-FL reduced RSV viral load in airway epithelial cells but not in PBMCs.

These in vitro and ex vivo data suggest that treatment with hMOS, including 2’-FL, 3’-SL, and 6’-SL, makes airway epithelial cells and PBMCs more resistant to RSV. These results are consistent with the observations that breastfed infants have a lower risk of severe RSV disease and with the clinical results linking 2’-FL and LNnT in infant formula with fewer respiratory infections. The mechanism whereby consumption of various hMOS compositions and concentrations causes breastfed infants to resist RSV infection and their mechanisms of action remain a promising topic of investigation.

### hMOS modulates gut microbiome to mitigate the severity of RSV infection

The proposition of a gut-lung axis suggests that gut microbiota contributes to the systemic immune defense against infectious and inflammatory diseases beyond the gastrointestinal tract, especially in the respiratory system [74]. Emerging evidence links alterations in the gut microbiome and its metabolites with the incidence and severity of respiratory infections, including RSV [75,76].

In a study that builds on the clinical trial by Puccio et al. [24] (2017), Berger et al. [77] (2020) measured the impact of 2’-FL and LNnT in infant formula on infant gut microbiome composition with an exclusively breastfed group added as reference. At 3 mo of age, 3 fecal community types (FCTs) were identified. Breastfed infants had a prevalence (∼60%) of FCT *Bifidobacteriaceae* at high abundance (FCT BiH), a bacterial cluster dominated by *Bifidobacterium*. FCT *Bifidobacteriacea* (FCT Bi), a more diverse cluster harboring not only *Bifidobacteriaceae* but also high concentrations of *Lachnospiraceae,* was present in 85% of the formula-fed control group*.* FCT *Enterobacteriaceae*, characterized by a high abundance of *Enterobacteriaceae* and *Lachnospiracea,* was observed in all groups without significant differences. Intriguingly, 40% of the infants who were given the 2’-FL and LNnT infant formula presented an FCT BiH microbiome, characteristic of the breastfed infants, whereas around 45% of the infants developed an FCT Bi microbiome, characteristic of the control group. Formula-fed infants with FCT BiH microbiota at 3 mo were less likely to require antibiotics ≤12 mo (OR: 0.4; 95% CI: 0.17, 0.93; *P* = 0.03) than those with FCT Bi or FCT *Enterobacteriaceae* [77]. The 2’-FL and LNnT promoting a *Bifidobacterium*-dominated microbiota in some infants but not in others suggests that maternal and infant factors may influence the ability of 2’-FL and LNnT to foster *Bifidobacterium*. For example, the infant Se phenotype is also a determinant of the *Bifidobacterium* species present in the feces [78], and different species of *Bifidobacteria* have distinct abilities to utilize hMOS [79]. Additionally, factors such as other components of formula and human milk, as well as the maternal microbiota, which seed the infant microbiota, may also be involved. A better understanding of these interrelated factors is requisite to the improvement of clinical outcomes through the modulation of the microbiota by hMOS.

Consumption of 2’-FL and LNnT and elevated fecal fucosylated glycans, lactate, acetate, and *Bifidobacterium* were the main features separating infants who experienced bronchitis or LRTIs from those who did not [80]. At 3 mo, healthy infants had a higher relative fecal abundance of *Bifidobacterium spp.*, *B. infantis*, and acetate and lower relative fecal abundance of propionate, butyrate, and 5-aminovalerate relative to infants who had bronchitis or LRTIs. Relative abundances of total *Bifidobacterium* as well as total *B. longum*, *B. longum* subsp*. longum* and *B. longum* subsp. *infantis* were positively correlated with the relative abundance of acetate in 3-mo-old fecal samples [80]. In an experimental model, LNnT and LNT significantly increased *B. longum* subsp. *infantis* growth and acetate production, whereas 2’-FL did not. The spent culture media from *B. longum* subsp*. infantis* that had contained LNT or LNnT strongly decreased nuclear factor kappa B activation by TNF-α [80]. Additionally, the spent culture media from *B. longum* subsp. *infantis* conditioned with LNnT, LNT, or 2’-FL significantly reduced *Salmonella* (SL1344) invasion in cultured Caco-2 cells. Moreover, the addition of acetate to the spent culture media of *B. longum* subsp. *infantis* grown on glucose led to a similar reduction in *Salmonella* invasion as spent culture media conditioned with LNT. Based on these data, the authors hypothesize that acetate is a key *Bifidobacterium* metabolite stimulated by specific hMOS, such as LNT and LNnT, that promote epithelial barrier function and protection against respiratory tract infections [80].

Shotgun metagenomic sequencing and untargeted mass spectrometry metabolomics on infant stool samples revealed that consumption of 2’-FL and LNnT promoted higher concentrations in *N*-acetylated-amino acids and gamma-glutamylated amino acids and reduced free amino acid content in feces [81]. Gamma-glutamylated amino acids were positively correlated with the presence of *Bifidobacterium bifidum*, *B. longum* subsp. *longum*, and *B. longum* subsp. *infantis* and negatively correlated with LRTI incidence during the first year of life [81]. The feces of infants consuming the unsupplemented control formula contained more sphingolipids, such as sphinganine and 3-keto-sphinganine, which were positively correlated with *Bacteroides* species and with LRTI incidence [81]. *N*-acetylated-amino acids and gamma-glutamylated amino acids produced by gut bacteria can be absorbed by the infant and hydrolyzed in the liver into acetate or glutamine and free amino acids, potentially contributing to circulating metabolites reaching other organs, such as the lungs, influencing the risk of LRTIs [81].

Other studies investigated associations between the gut microbiome and respiratory infections without studying hMOS. The early microbiome associated with C-section has significantly lower *Bifidobacterium* and higher *Klebsiella* and *Enterococcus* abundances and is associated with a higher cumulative incidence of respiratory infections over the first year of life [82]. A case-control study compared the gut microbiome at 3 mo of age from 40 infants hospitalized with bronchiolitis (65% positive for RSV) with 115 healthy controls [83]. Through an unbiased approach, 4 distinct fecal microbiota profiles emerged. Compared with infants who had the *Enterobacter*/*Veillonella*-dominant profile, those with the *Bacteroides*-dominant profile had a greater likelihood of bronchiolitis (OR: 4.24; 95% CI: 1.56, 12.0; *P* = 0.005) [83].

To investigate whether severe RSV disease is associated with a distinctive gut microbial profile, Harding et al. [84] (2020) compared the gut microbiome of 58 infants hospitalized with confirmed RSV infection (of which 5 had severe RSV disease and were admitted to a pediatric intensive care unit) with 37 healthy controls. At 72 h after hospitalization, the overall microbiome composition was significantly different between RSV-infected and control infants as well as between infants with moderate and severe RSV disease; distinctive groups included the genera *Clostridiales*, *Lactobacillus*, *Oscillospira*, *Odoribacter*, *Tissierella Soehngenia*, and S24_7 [84]. Healthy controls had a significantly higher relative abundance of *Lactobacillus* OTU 59. RSV-infected infants had a significantly higher abundance of *Lactobacillus* OTU 16. Also, infants with RSV infection had a lower abundance of *Tissierela Soehngenia* and a higher abundance of *Odoribacter* and S24_7 operational taxonomic units than controls. Moreover, infants with severe RSV had an even higher abundance of *Odoribacter* and S24_7 operational taxonomic units than those with moderate disease [84]. Some S24_7 members can degrade IgA, which could weaken mucosal defense and contribute to RSV disease severity. An increase in S24_7 abundance was observed in the gut microbiome after lung infection with RSV or H1N1 influenza A in mice [85,86]. A fruitful area of investigation would be to determine whether these differences in the infant gut microbiome composition are a cause or a consequence (or both) of RSV infection.

Animal and mechanistic studies both provide information on how gut microbiota modulate the immune response against RSV infection. A high-fiber diet protects mice from weight loss after RSV infection and reduces viral load as well as the number of inflammatory cells in the lungs relative to a low-fiber diet and control [87]. Mice fed the high-fiber diet presented a higher abundance of *Lachnospiraceae* and higher acetate concentrations in the lumen of the gut, which correlated with a reduced RSV viral load in the lungs [87]. These protective effects were abolished by treatment with antibiotics before RSV infection, indicating an essential role of the microbiota in the protection against RSV disease. Remarkably, direct supplementation of acetate to mice simulated the protection generated by a high-fiber diet through the induction of interferon beta (IFN-β) in the lung mediated by G-protein-coupled receptor 43 and IFN type 1 receptors [87]. Another study showed that an oral intervention with probiotics in mice decreased RSV-induced lung inflammation through IFN-β expression mediated by acetate [88]. To investigate the role of acetate on human RSV disease, Antunes et al. [89] (2022) collected fecal samples from 17 infants with RSV bronchiolitis within 48 h of hospitalization. A significant positive association existed between *Dysgonomonadaceae* and acetate in stool. *Bacteroidaceae* was positively associated with length of hospitalization and a longer duration of cough and nasal congestion. High concentrations of acetate were significantly associated with better oxygen saturation at admission and fewer days of fever. In cells from nasopharyngeal washes of infants with RSV bronchiolitis, treatment with acetate reduced viral load and increased expression of antiviral genes [89]. Thus, specific infant gut microbiota that produce acetate attenuate symptoms and reduce the severity of RSV bronchiolitis. However, no relationship was reported between breastfeeding and fecal acetate concentrations in this study of infants with moderate and severe bronchiolitis, but regardless of the mechanism, breastfeeding is known to be protective against RSV. The associations between higher fecal *Bifidobacterium* abundance and acetate concentration with lower LRTI risk in infants receiving 2’-FL and LNnT support further studies on the strength and mechanisms of defense against RSV by hMOS.

### Future directions

**FIGURE 3:**
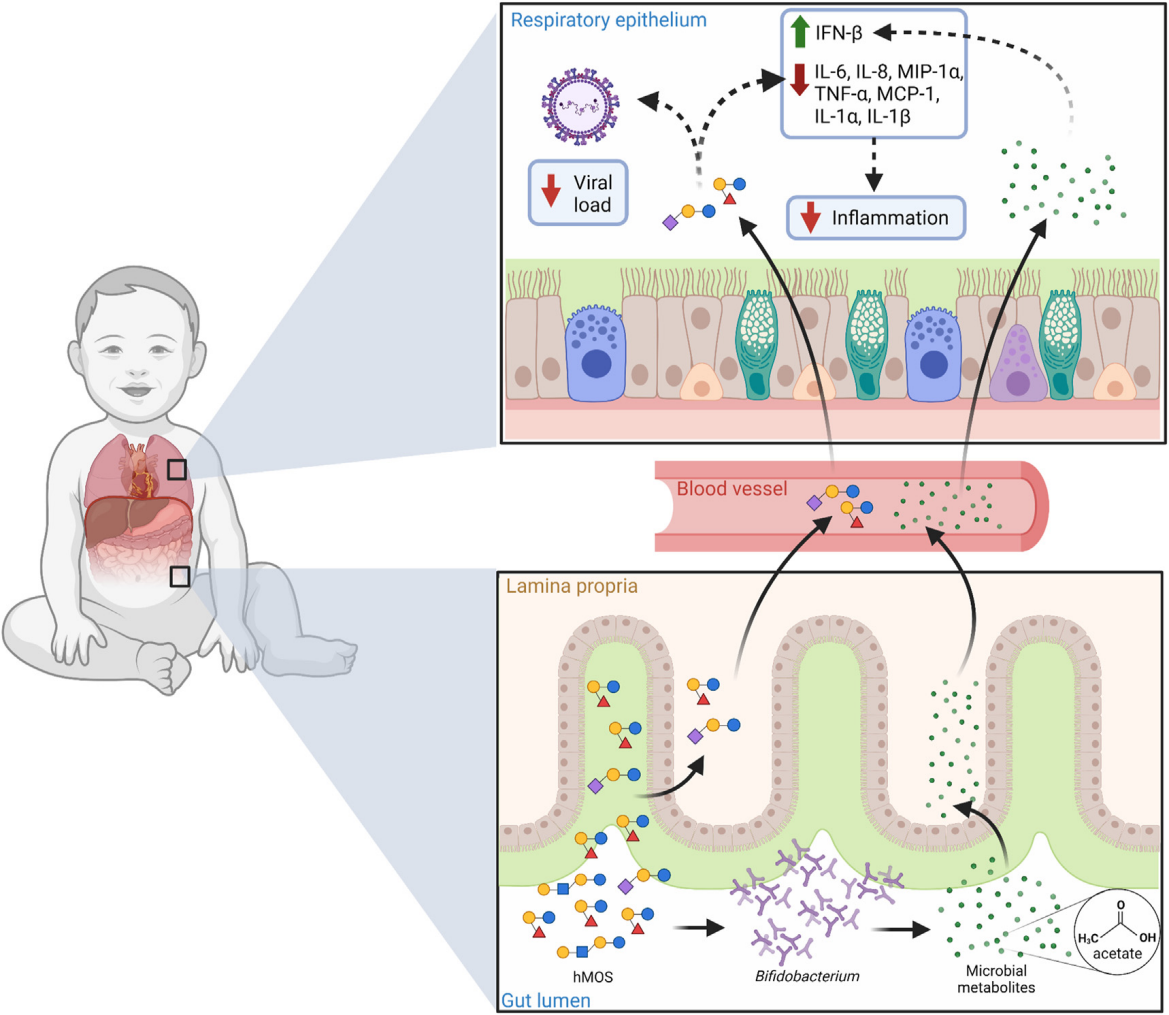
Potential role of hMOS on RSV disease. hMOS such as 2’-FL and LNnT are metabolized by *Bifidobacterium* in the infant’s gut into short-chain fatty acids, like acetate. Small quantities of hMOS and acetate are absorbed and can reach the lungs through circulation, where they could act as antivirals and modulate inflammation. Small quantities of hMOS and short-chain fatty acids could also coat the upper respiratory mucosa in the form of regurgitated milk, as seen in the noses of breastfed infants. hMOS, human milk oligosaccharides; IFN-β, interferon beta; IL-1α, interleukin-1 alpha; IL-1β, interleukin-1 beta; IL-6, interleukin 6; IL-8, interleukin 8; LNnT, lacto-N-neotetratose; MCP-1, monocyte chemoattractant protein-1; MIP-1α, macrophage inflammatory protein-1 alpha; RSV, respiratory syncytial virus; TNF-α, tumor necrosis factor alpha; 2’-FL, 2’-fucosyllactose.

hMOS shows promise in combating RSV infection and disease through the circulation (a gut-lung axis). Direct effects include antiviral action and immunomodulation in the intestinal mucosa. Modulation of the gut microbiota and its metabolites, in turn, could have indirect effects on the mucosa of the respiratory tract and also affect immune cell function throughout the body (Figure 3). Small quantities of hMOS and short-chain fatty acids could also have a direct effect by coating the upper respiratory mucosa in the form of regurgitated milk, as seen when milk exits the noses of breastfed infants. The current data from clinical and experimental studies that connect hMOS to modulation of the immune response, composition and function of gut microbiome, and reduced risk of LRTIs raise new questions and justify more comprehensive investigations on mechanisms whereby hMOS reduces the incidence of or ameliorates the intensity of RSV infection.

Most human studies published to date were conducted with infants who were hospitalized, that is, on the most severe RSV disease cases. Why does RSV go unnoticed in most infants, whereas some require pediatric intensive care unit care with high mortality risk that may involve lack of human milk? Breastfeeding lowers the risk of severe RSV disease. However, breastfed infants are not a homogenous group. In addition to the variation of infant genes, variation of maternal genes causes human milk to harbor a multitude of highly variable bioactive components. These many variable factors are undoubtedly interrelated in the overall risk of RSV infection and severity. Future studies should include human milk composition, RSV-confirmed symptomatic and asymptomatic infants, infant and maternal metagenomics, gut microbiota genomics and metabolomics, and measures of immune modulation, such as nuclear factor kappa B activation, and expression of IL-6, IL-8, macrophage inflammatory protein-1 alpha, TNF-α, monocyte chemoattractant protein-1, interferon gamma-induced protein 10, IL-1α, IL-1β, and IFN-β. Defining their interactions on the incidence and severity of RSV infection and disease outcomes may inform future prophylaxis and treatment.

One essential step toward that end is to develop standardized and validated methods for the identification and absolute quantification of human milk components, including hMOS, to provide reliable and comparable data between studies and among populations. Most of the various research groups use different extraction techniques and analytic methods for hMOS that contribute to variability and compromise the interpretation of data across studies [33].

Most current data about hMOS and respiratory infections come from clinical studies originally designed to investigate the safety of infant formulas that contain hMOS. Therefore, the secondary analysis of these data for hMOS effects on respiratory infections and RSV infection is limited by the experimental design. Optimizing study design for hMOS effects on RSV disease should be considered in future studies. The dependence on maternal recall or medical records can bias the measurement of disease incidence and severity; future studies should provide a direct assessment of the infants while they have active symptoms, as well as measure asymptomatic infections. Respiratory disease definitions should be standardized. The severity of symptoms and more fully defining the pathogens that underlie the symptoms would provide data for investigating mechanisms of protection. One should also consider investigating the possibility of hMOS having a stronger effect on preventing reinfection and long-term RSV-mediated atopic asthma than primary infection.

In this context, large, community-based, prospective birth cohort studies are best able to verify the current evidence on the contribution of hMOS toward the prevention of respiratory infections and RSV disease in infant populations. These studies may also identify other human milk bioactive components associated with RSV disease outcomes not yet tested in experimental and clinical studies. The gut-lung axis is a 2-way street. Prospective stool sampling starting in the first weeks of life, to encompass before, during, and after RSV infection, with shotgun metagenomic analysis to increase bacterial taxa resolution, could elucidate whether changes in the microbiome precede or follow RSV infections and allow inferences regarding causality.

Many open questions remain for future studies: Are specific hMOS protective against RSV disease in infants? By what mechanisms do they protect? Are breastfed infants protected from severe RSV disease through microbiota-produced acetate or other organic acids induced by dietary hMOS? Such studies may help to identify hMOS with prophylactic or therapeutic action against RSV, as well as identify the most vulnerable infants to severe RSV disease based on their measurable risk factors. Such advances would be a powerful addition to the RSV vaccine now available to infants at risk.

## Author contributions

The authors’ responsibilities were as follows – ALM, DSN: design; KMT: writing; KMT, SC, ALM, DSN: final content; and all authors: read and approved the final manuscript.

## Conflict of interest

The authors report no conflicts of interest.

## Funding

The authors reported no funding received for this study.
